# Noninvasive radiomic analysis of enhanced CT predicts CTLA4 expression and prognosis in head and neck squamous cell carcinoma

**DOI:** 10.1038/s41598-023-43582-0

**Published:** 2023-10-05

**Authors:** Yeping Zhu, Mianhua Wu

**Affiliations:** Nanjing University of Chinese Medicine, No. 282, Hanzhong Road, Nanjing, Jiangsu China

**Keywords:** Cancer, Biomarkers, Medical research

## Abstract

Developing a radiomic model to predict CTLA4 expression levels and assessing its prognostic accuracy for patients. Medical imaging data were sourced from the TCIA database, while transcriptome sequencing data were derived from the TCGA database. We utilized a linear kernel SVM algorithm to develop a radiomic model for predicting CTLA4 gene expression. We then assessed the model’s clinical relevance using survival and Cox regression analyses. Performance evaluations of the model were illustrated through ROC, PR, calibration, and decision curves. (1) Bioinformatics analysis: Kaplan–Meier curves indicated that increased CTLA4 expression correlates with enhanced overall survival (OS) (p < 0.001). Both univariate and multivariate analyses revealed that high CTLA4 expression served as a protective factor for OS (HR = 0.562, 95% CI 0.427–0.741, p < 0.001). (2) Radiomics evaluation: the ROC curve demonstrated that the AUC for the SVM radiomics model was 0.766 in the training set and 0.742 in the validation set. The calibration curve affirmed that the model's prediction probability for high gene expression aligns with the actual outcomes. Furthermore, decision curve analysis (DCA) indicated that our model boasts robust clinical applicability. CTLA4 expression level serves as an independent prognostic factor for HNSCCs. Using enhanced CT images, the SVM radiomic model effectively predicts CTLA4 expression levels. As a result, this model offers strong prognostic insights for HNSCCs, guiding precise diagnosis, treatment, and assisting in clinical decision-making.

## Introduction

Head and neck squamous cell carcinoma (HNSCC) originate from the mucosal epithelium of the oral cavity, pharynx, comprising approximately 90% of all the head and neck tumors^1^. It is the seventh most common type of cancer worldwide, with about 930,000 new cases and 470,000 deaths in 2020^2,3^. The incidence of HNSCCs continues to rise and is estimated to increase by 30%, that is, 1.08 million new cases annually, by 2030 (Global Cancer Observatory)^4,5^. Numerous studies have reported a close relationship between HPV status and the prognosis of HNSCC. HPV positivity often manifests as poorly differentiated cancer, early lymphatic metastasis and late staging, but is sensitive to radiotherapy and chemotherapy^6,7^. Currently, the primary treatment for HNSCC involves a bombination of surgery, radiotherapy and chemotherapy. However, the 5-year survival rate is less than 50%, which is alarmingly low^8^. From the clinical perspective, accurate prognostic indicators are crucial for the effective treatment and management of HNSCC^9^. Tthe predominant method for prognostic evaluation of HNSCC is the American Joint Committee on Cancer (AJCC) tumor-node-metastasis (TNM) staging system^10,11^. However, it is limited in scope due to insufficient anatomical and biological prognostic factors, as well as its reliance on experience of individual clinician. Given this context, the current methods often fall short in providing the most precise treatment plans, contributing to the low survival rates for HNSCC patients. There is a pressing need for newer, more robust prognostic evaluation techniques and indicators to enhance individualized precision treatment.

Cytotoxic T-lymphocyte associated protein 4(CTLA4) gene, a member of the immunoglobulin superfamily, serves as a negative immune regulator and is constitutively expressed on regulatory T (Treg) cells^12^. Traditionally, mutations in this gene have been associated with autoimmune diseases^13–15^. In recent years, studies have found that mutations of this gene are closely related to malignant tumors such as lung, bladder and head and neck cancers^16–18^. Data from ClinicalTrials.gov indicates that there are 23 clinical trials exploring treatments for HNSCC by inhibiting CTLA4 expression. Therapies that target T cell inhibitory checkpoint proteins like CTLA4 and programmed cell death-Ligand 1 (PD-L1) have shown efficacy for various cancers, including HNSCCs. Ipilimumab, currently the sole FDA-approved CTLA4 inhibitor, promotes massive T cell proliferation and combats tumor cells by preventing CTLA4 from binding to its B7 molecular ligand^19,20^. Nivolumab is a blocking antibody against human programmed death receptor-1(PD-1), which can suppress tumors through PD-1-mediated immune response^21^. In the clinical trial nct02741570^22^, the combination of nivolumab and ipilimumab extended the overall survival (OS) by 3.0 months compared to the cisplatin/carboplatin group.

Radiomics is a mathematical-statistical procedure that extracts and quantifies high-throughput features from medical images, facilitating noninvasive profiling of tumor heterogeneity^23^. The technology offers a dynamic and non-invasive approach to detect and quantitatively assess tumor attributes. Radiomics technology has been widely used in clinics^24^. Existing research demonstrates its potential in early diagnosis, classification of HNSCC, and in evaluating tumor heterogeneity and its microenvironment^25–27^. Currently, most methods for detecting CTLA4 based on biopsy samples are invasive, due to the spatiotemporal heterogeneity of tumors, it is difficult for a single local tissue detection result to represent all tumor tissues well. And artificial detection through immunohistochemistry is time-consuming and labor-intensive, it is difficult to ensure the consistence and the results are influenced by various factors such as platforms, reagents, and researchers and so on. Considering the above factors, we have chosen radiomics information. Yet to date, there haven’t been any radiomic studies specifically exploring CTLA4 in HNSCC.

To summarize, this study pioneers the non-invasive prediction of the CTLA4 expression in HNSCC using CT radiomics. We also evaluated the correlation between the constructed radiomics model and related genes and prognosis. By harnessing bioinformatics, we delved into the potential molecular mechanisms underlying CTLA4 expression and its relationship with the immune microenvironment.

## Methods

### Patient cohort and data collection

Transcriptome sequencing data for a total of 528 HNSCC cases, along with clinical and follow-up data, were downloaded from The Cancer Genome Atlas (TCGA). (https://portal.gdc.cancer.gov/). Samples missing complete clinical information, those with survival times less than 30 days, or those that were not solid tumors or lacked sequencing data were excluded from the study.

In addition, 211 cases featuring enhanced CT arterial phase were obtained from The Cancer Immunome Atlas (TCIA) database (https://www.cancerimagingarchive.net/). We excluded samples that were postoperative, had inferior image quality, or were devoid of sequencing data. We defined the following situations as poor image quality: There were artifacts in the image, such as motion artifacts, metal radial artifacts caused by dentures; missing image or incomplete scanning of tumor site.

**Figure 1:**
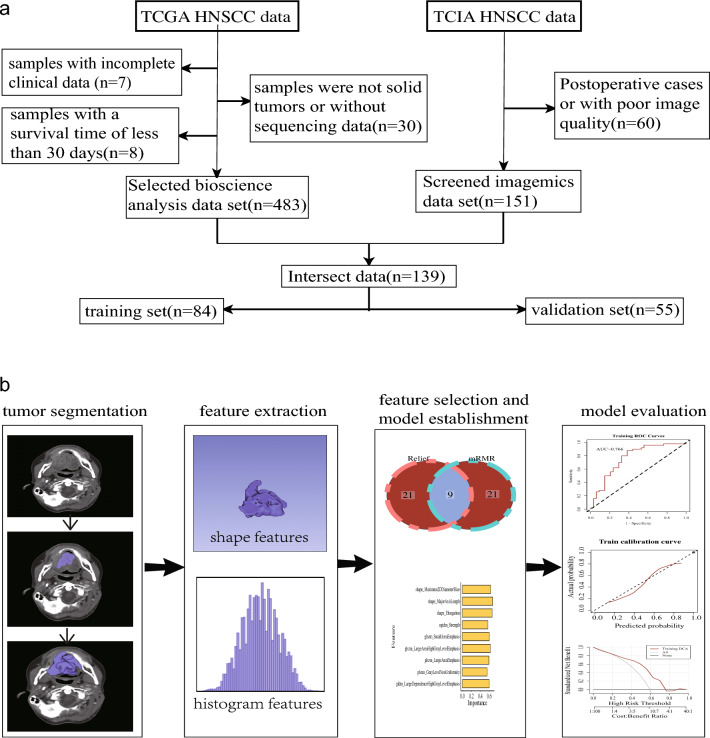
The flowchart of data collection and analysis. (**a**) Data screening process and dataset division. (**b**) Brief flowchart of radiomic progression. *TCGA* The Cancer Genome Atlas, *TCIA* The Cancer Imaging Archive, *HNSCC* Head and neck squamous cell carcinoma.

All the data and images have been anonymized and are publicly available, so exemption ethics and informed consent. The brief flowchart is shown in Fig. 1.

### Bioinformatics

#### Data preprocessing

We used UCSC XENA (https://xenabrowser.net/datapages/) RNAseq data in TCGA's FRKM format processed uniformly through the Toil process (Vivian Jet al., 2017). Then, we extracted RNAseq data from HNSCC tissues and normal tissues of TCGA. And converted the RNAseq data in FRKM format into log2, compared the expression between samples by the Xiantao Tool (https://www.xiantao.love/products). The cutoff values for CTLA4 molecule expression were determined based on the "survminer" R package, and patients were divided into two groups based on high and low CTLA4 expression.

#### Survival data analysis

In this study, overall survival (OS) is defined as the duration from randomization until any factor resulting in patient death^28^. The OS rates of the patients in both groups were assessed through Kaplan–Meier analysis. Both univariate and multivariate Cox proportional hazard regression analyses were performed to examine the determinants of OS. This also facilitated the evaluation of the prognostic significance of different variables such as CTLA4 expression level, age, gender, tumor grade, perineural invasion, therapy, primary tumor site, HPV status.

#### Gene set variation analysis

Using the GSVA (Gene Set Variation Analysis) package of R, we calculated the expression matrix of patients with head and neck squamous cell carcinoma, as well as the pathway enrichment scores of KEGG and hallmark gene sets in each sample. The enrichment scores of CTLA4 high and low-expression groups were analyzed by using R-package "limma". The top 15 differential pathways were visualized. The “phatmap” package was utilized to generate the heatmap. This heatmap served as a foundation for unsupervised classification, allowing us to determine if samples with similar pathway activities held distinct biological significance.

#### Correlation analysis

To analyze the correlation with rumor clinical features, we used the "corrplot" package in R. Spearman's rank correlation coefficient was used to assess the relationship between the primary variable, CTLA4, and the clinical characteristics of the tumors.

For the analysis of immune cell infiltration, we uploaded the gene expression matrix to the cibersortx database (https://cibersortx.stanford.edu/) using the "corrplot" package in R. This facilitated the computation of immune cell infiltration for each sample. Again, Spearman's rank correlation coefficient was utilized to discern the correlation between the main variable CTLA4 and immune cell infiltration. The findings were presented through a correlation heatmap.

### Radiomics analysis

#### Image preprocessing and segmentation

The data was preprocessed using Z-Score standardization. The images were resampled with the same voxel size (1 × 1 × 1 mm^3^) to minimize the variation arising from differences in scanning equipment, protocols, and the scale of patients' lesions.

3D slicer software (version 4.10.2; https://www.slicer.org/) was used for 3D volumes of interest (VOIs) segmentation. Tumor segmentation was conducted by reader 1 (with 5 years of experience in CT), and 30 randomly selected samples were confrmed again by reader 2 (with 8 years of experience in CT). Both readers were blinded to clinical information and pathological results.

#### Radiomic feature extraction

Feature extraction was performed using Python's open source pyradiomics package, a total of 107 features were extracted including first-order features, volume and shape features, texture features and wavelet-transformed features (Supplementary Tab. S1 for details).

#### Feature selection

For feature selection, two main steps were applied:

1. Intraclass correlation coefficient (ICC) is used to evaluate the consistency of radiomics features extracted based on two doctors' respective segmentation. It is generally believed that ICC ≥ 0.8 means good consistency, 0.51 ~ 0.79 means medium, and less than 0.50 means poor^29^. Select the features with ICC ≥ 0.8 to enter the next feature screening.
2. Max-Relevance and Min-Redundancy (mRMR)^30^ and Relevant Features (Relief) are performed to filter common features with "mRMRe" package and "FSelector" package in R.

The first 30 features selected by mRMR method and the first 30 features selected by the relief algorithm are intersected, 9 features were obtained as follows: original_shape_Maximum2DDiameterSlice, original_shape_Elongation, original_shape_Major Axis Length, original_glszm_Large Area Emphasis, original_gldm_Large Dependence High Gray Level Emphasis, original_glszm_Small Area Emphasis, original_glszm_Large Area High Gray Level Emphasis, original_glszm_Gray Level NonUniformity and original_ngtdm_Strength.

#### SVM Model construction and evaluation

The Support Vector Machines (SVM) is a binary classification model that operates on the principle of finding the best hyperplane in the feature space to separate data points of different classes. It aims to maximize the margin between the two classes and uses support vectors to define the decision boundary. In our study, we utilized the selected radiomics features to construct an optimal SVM model. To achieve this, we employed a grid search method with fivefold cross-validation. This approach allowed us to systematically explore different combinations of hyperparameters to find the best-performing SVM model with the highest accuracy in classifying the data into the two groups based on the CTLA4 expression level. The final values used for the model were cost = 0.125 and weight = 1.

The probability of predicting gene expression level by the output of radiomcis model is defined as rad-score (Radiomics Score, RS). We determined the cutoff value of RS using the 'survminer' R package and divided patients into two groups based on high and low RS. Then the RS of SVM radiomics model was combined with clinical data, and the p-value of each variable in the analysis of differences between groups was > 0.05, indicating that the baseline situation of patients with high and low RS was close and comparable.

#### Statistical analysis

R software (version 3.3.3) was used for statistical analysis and model building. The Standardized variables were expressed as mean ± standard deviation (SD) or as median (25 quantile, 75 quantile), and evaluated by Shapiro–Wilk test, independent sample t-test, Mann–Whitney U test and chi-squared test.

Additionally, Kaplan–Meier analysis, Log-rank test, Wilcoxon test, Spearman correlation analysis, univariate logistic regression analysis, univariate and multivariate Cox regression analysis were used for survival analysis. Likelihood ratio test was used to analyze the interaction between CTLA4 and other covariates. A P-value < 0.05 was considered statistically significant.

The evaluation index of the SVM model is Area Under Curve (AUC), accurate (ACC), specificity (SPE), sensitivity (SEN), positive predictive value (PPV) and negative predictive value (NPV). Delong test was used to compare the AUC values of the training set and validation set. ROC curves were plotted with the “pROC” package. Calibration curves were performed by the “rms” package. DCA curves were done by the function of “dca. R.”

The calibration curve compares the difference between predicted values and actual results by drawing an ideal straight line (45 degree angle line). If the calibration curve is close to the ideal straight line, it indicates that the prediction results of the model are accurate and reliable. If the calibration curve deviates from the ideal straight line, then there is a deviation in the model. In the DCA curve, ALL and None are two reference lines, with benifit as the vertical axis and different risk thresholds as the horizontal axis. When the curve is located above ALL and None as two reference lines, it indicates that the model used can improve clinical net benefits within the corresponding threshold range.

### Ethics declarations

All the data and images were anonymized and are publicly available, so exemption ethics and informed consent.

## Results

### Baseline data and patient characteristic

For the genomics dataset, 483 HNSCC cases from the TCGA database were split based on CTLA4 expression into a high expression group (n = 282) and a low expression group (n = 201) with a cutoff value of 1.1909 (determined through the “survminer” R). The dataset covered 128 males and 355 females. There was no significant difference in the distribution of age, gender between high and low CTLA4 expression groups (p = 0.873).

For the radiomics dataset, data were randomly split into a training set and a verification set at a 6:4 ratio (Training set 84 cases; Validation set 55 cases) (Supplementary Tab. S2). Each variable’s p-value was > 0.05 in the inter-group difference analysis , indicating comparable baselines for the training and vadidation sets.

### Bioinformatics

#### Differential gene expression

**Figure 2:**
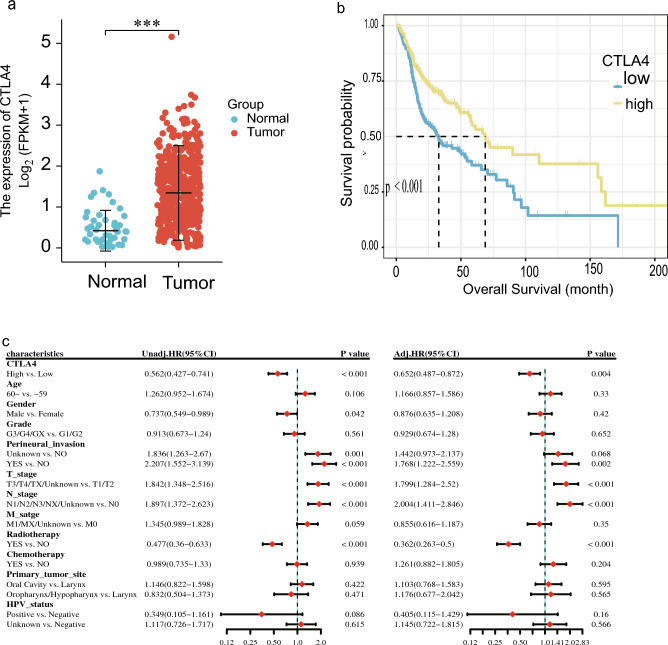
The comparison of clinical and survival data. (**a**) Comparison of CTLA4 expression level between HNSCC tissue and normal tissue. "***", "**" and "*" indicate the P values of 0.001, 0.01 and 0.05, respectively. (**b**) The CTLA4 expression level Kaplan–Meier curve shows that patients with high expression level have longer overall survival (OS) than patients with low expression. (**c**) Forest map of univariate and multivariate cox proportional-hazards model regression analysis.

CTLA4 expression in tumors was significantly higher than that in normal tissue (P < 0.05). The median difference between the two groups was 0.852, and the difference was statistically significant (P < 0.001) (see Fig. 2a).

#### Survival analysis

The median survival time of CTLA4 low expression group was 32.83 months (95% CI 22.5–54.7), while the high expression group had a median survival time of 68.8 months (95% CI 57.73–158.666). KM curve indicated that increased CTLA4 expression correlated with improved OS (p < 0.001) (Fig. 2b).

In univariate analysis, high CTLA4 expression was a protective factor for OS (HR = 0.562, 95% CI 0.427–0.741, p < 0.001). After multivariate adjustment, it remained a significant protective factor (HR = 0.652, 95% CI 0.487–0.872, p = 0.004) (Fig. 2c).

In the subgroup analysis by age, increased CTLA4 expression was a protective factor for OS in both subgroups: those under 60 years (HR = 0.495, 95% CI 0.316–0.775) and those over 60 years (HR = 0.612, 95% CI 0.43–0.867). The interaction test p-value was 0.47, suggesting no significant interaction between CTLA4 expression and age subgroups. This indicates that the impact of CTLA4 on OS is consistent across age groups.

#### Correlation analysis

Correlation analysis of tumor clinical features revealed that CTLA4 expression significantly correlated with T stage (p < 0.001), tumor grade (p < 0.01), M stage (p < 0.01), primary tumor site (p < 0.01), HPV status (p < 0.05) and radiotherapy (p < 0.05).

The immune cell infiltration correlation heatmap indicates a significant correlation between CTLA4 and T cells CD8 (p < 0.001).

#### GSVA

In the KEGG gene set analysis, pathways enriched in the CTLA4 high expression group included the p53 signaling pathway, VEGF signaling pathway, and mTOR signaling pathway. Similarly, in the hallmark gene set, the CTLA4 high expression group showed enrichment in the p53 pathway and apoptosis pathway.

### Radiomics analysis

#### Model evaluation and verification

**Figure 3:**
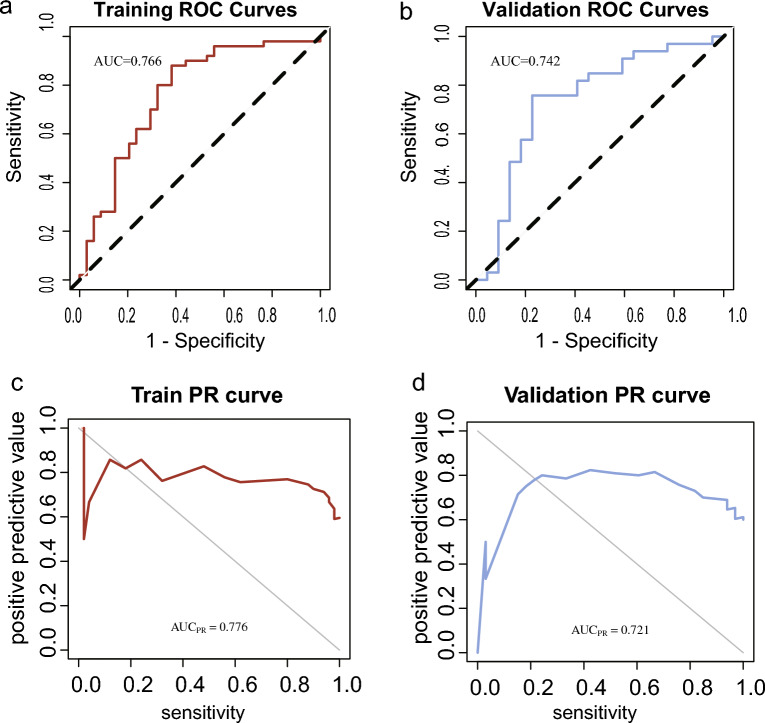
ROC curves and PR curves of the radiomic model. (**a**) Receiver operating characteristic curve of the model in the training set. (**b**) Receiver operating characteristic curve of the model in the validation set. (**c**) Precision recall (PR) curve of the model in the training set. (**d**) Precision recall curve of the model in the validation set.

The ROC curves presented in Fig. 3a,b show an AUC value of 0.766 for the training set and 0.742 for the validation set. Moreover, the Precision-Recall (PR) curves in Fig. 3c,d for both sets predominantly arch towards the upper right corner, indicating good model performance.

**Figure 4:**
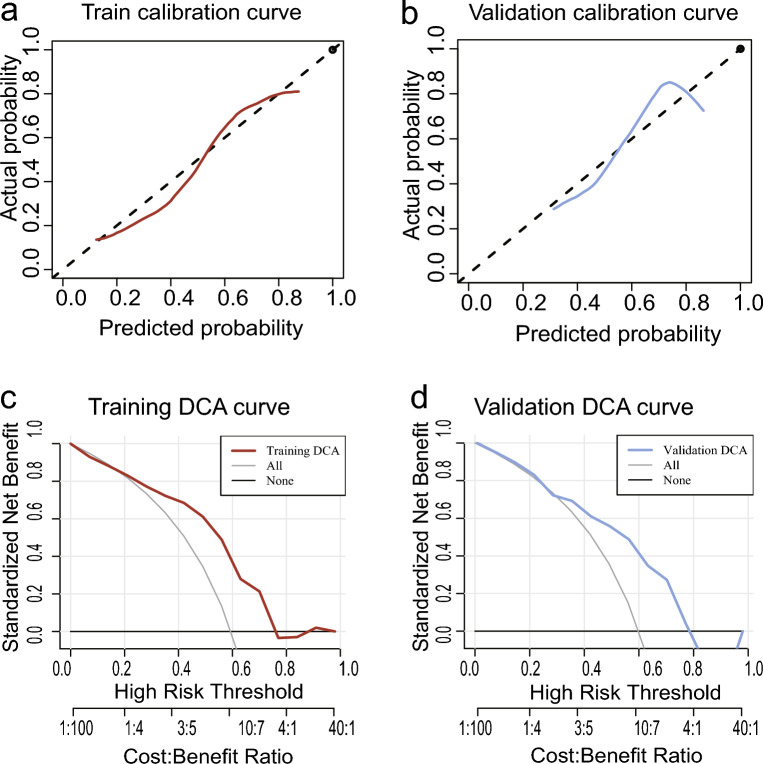
Calibration curves and decision curve analysis of the radiomic model to evaluate model prediction efficiency. (**a**) Calibration curve of the model in the training set. (**b**) Calibration curve of the model in the validation set. (**c**) Decision curve of the model in the training set. (**d**) Decision curve of the model in the validation set.

Furthermore, calibration curves were used to evaluate the calibration of the nomogram between predicted outcome events and real events. In a well-calibrated model, the prediction will fall on the diagonal of 45-degree. As shown in Fig. 4a,b, both in training set and validation set, the prediction of outcome events was in good agreement with the actual observation.

Within a threshold range of 0.1–0.6, the DCA curve surpasses both the None and All curves, suggesting the model offers significant clinical utility. The AUC values between the training and validation sets showed no significant difference (p = 0.801), indicating a consistent and well-fitted model (see Fig. 4c,d).

**Figure 5:**
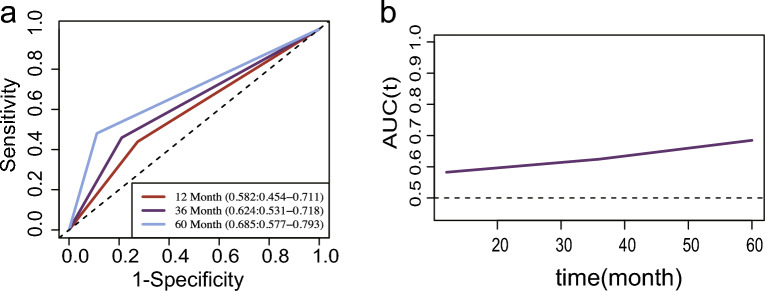
Time dependent curve of RS (Radiomics score). Time dependent ROC curve of RS, assessing different degree of RS in predicting patient survival at different time nodes.

As shown in Fig. 5, the AUC of nomogram time-dependent ROC for 12-month, 36-month, and 60-mont was 0.582, 0.624, and 0.685, respectively, indicating an upward trend.

#### Analysis of differences between groups

**Figure 6:**
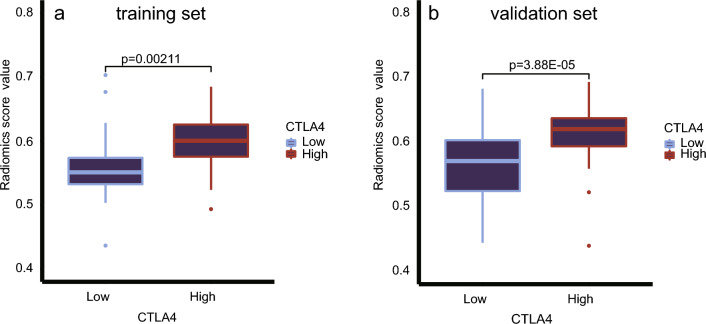
A comparison of RS between groups of high and low CTLA4 expression in training set (**a**) and validation set (**b**).

In the training set, the Radiomics Score (RS) distribution significantly differed between the high and low CTLA4 expression groups (P < 0.001), with the high-expression group showing elevated RS values. This trend was consistent in the validation set (P = 0.002) (see Fig. 6).

#### Correlation analysis

The RS's correlation with immune genes was assessed^31^, selecting genes with a correlation P-value < 0.05 for visualization. Notably, RS exhibited a positive correlation with CTLA4, evidenced by a correlation coefficient of 0.36 (P < 0.001) (Supplementary Fig. S1).

#### Survival analysis

The Kaplan–Meier curve showed that a higher RS significantly correlated with better survival outcomes (P = 0.006). Specifically, The median survival time was 24.63 months (95% CI 22.5–54.7) for the low expression group and 72.2 months (95% CI 22.5–54.7) for the high expression group.

In the univariate analysis, it was found that high RS was a statistically significant protective factor for overall survival (HR = 0.463, 95% CI 0.262 − 0.816, P = 0.008). The significance persisted in the multivariate analysis, even after adjusting for other factors (HR = 0.365, 95% CI 0.193–0.691, P = 0.002).

## Discussion

HNSCC exhibits remarkably heterogeneity despite it develops from a single cell type in one tissue^32^. Many factors including immunity can affect the prognosis of HNSCC, and currenly, only few biomarkers can be used to accurately predict the prognosis. Thus, augmenting prognostic information can be valuable in guiding clinical decision-making^33^.

CTLA4 acts as a negative immune regulator by transmitting inhibitory signals to T cells, thus suppressing their activation and dampening the immune response. Recently, CTLA4 has been extensively studies in the context of cancer immunotherapy. Antibody drugs targeting CTLA4 have shown promising results in the treatment of various solid tumors, leading to their approval for clinical use. Additionally, the expression level of CTLA4 has been associated with prognosis and response to immunotherapy in different cancer types. Non-invasive methods to predict CTLA4 expression, such as radiomics, hold significant potential for guiding personalized treatment decisions and advancing precision medicine approaches in oncology^34–39^.

Our study is the first attempt to establish a correlation between RS and CTLA4 expression in HNSCC, shedding light on the potential of radiomics in predicting CTLA4 levels. CTLA4 is a prominent molecule in tumor immunotherapy, and antibody drugs targeting CTLA4 have emerged as a promising direction for anti-tumor therapy. The non-invasive nature of radiomics-based prediction of CTLA4 expression holds significant promise for personalized clinical decision-making. As we enter the era of big data and witness the advancements in radiomics, along with the growing demand for precision medicine, the integration of radiomics with genomics and proteomics is set to become a new and exciting research frontier.

In this study, we specifically investigated HNSCCs and conducted Kaplan–Meier analysis, confirming that patients with higher CTLA4 expression experience better prognoses (P < 0.001). Univariate and multivariate analyses consistently revealed that CTLA4 serves as an independent protective factor for HNSCC. Additionally, we examined the differences in imaging characteristics between patients with high and low CTLA4 expression and developed a radiomics prediction model for CTLA4 expression. These findings contribute to a better understanding of CTLA4's role in HNSCC and highlight the potential of radiomics in predicting CTLA4 expression levels, offering valuable insights for precision medicine in HNSCC treatment.

The comparison of AUC values in both the training set and validation set indicated no statistical difference (P = 0.801), indicating a good fit of the model. The calibration curve further confirmed that the model's predicted probabilities for high gene expression were in line with the actual observations. Moreover, the Time dependent ROC curve revealed that the AUC value increased with time, suggesting an improvement in the model's prediction efficiency over time. These findings support the reliability and potential clinical applicability of our radiomics prediction model for CTLA4 expression in HNSCC, indicating its usefulness in personalized prognostic assessment and treatment decision-making.

The comprehensive analysis of various evaluation metrics, including ROC curve, PR curve, validation curve, and DCA curve, supports our speculation that the radiomics model exhibits excellent predictive efficiency and holds clinical practicability. The transformation of CT-derived radiomic features into the quantitative Rad-score (RS) enabled us to establish an independent predictor for CTLA4 expression in HNSCC. Notably, the RS showed a significant correlation with HNSCC disease overall survival (HR = 0.463, 95% CI 0.262–0.816), indicating its potential as a valuable prognostic indicator in HNSCC. These findings highlight the importance of radiomics as a non-invasive, reliable method for predicting CTLA4 expression levels and its clinical implications in guiding personalized treatment decisions for HNSCC patients.

This study has some limitations that could guide future research. First, the sample size was relatively small, and further validation with larger cohorts is warranted. Second, the use of data from public databases may introduce some variability and bias, even though efforts were made to standardize the data. Future studies could consider collecting data from multiple centers to enhance the robustness of the findings. Third, manual lesion delineation could introduce human bias, and exploring semi-automatic or automatic methods for feature extraction could mitigate this issue. Finally, more advanced statistical methods could be employed to further enhance the accuracy and reliability of the predictive model. Moving forward, multi-center data validation and multi-modal imageomics research will be explored to address these limitations and improve the clinical utility of the radiomics model.

## Conclusion

In conclusion, CTLA4 expression level serves as a significant and independent prognostic factor for HNSCC (HR = 0.652, 95% CI 0.487–0.872). The SVM radiomics model, based on enhanced CT images, demonstrates its effectiveness in accurately predicting CTLA4 expression levels and exhibits a strong predictive power for HNSCC prognosis. Leveraging this model can aid clinicians in making informed clinical decisions and facilitate individualized and precise diagnosis and treatment strategies for HNSCC patients.

### Supplementary Information

Supplementary Figure S1.

Supplementary Table S1.

Supplementary Table S2.

Supplementary Legends.

## Data Availability

The data that support the findings of this study are available from TCIA (https://www.cancerimagingarchive.net) and TCGA (https://portal.gdc.cancer.gov).
