# Supplementary material for: Noninvasive radiomic analysis of enhanced CT predicts CTLA4 expression and prognosis in head and neck squamous cell carcinoma
Source: Sci Rep. 2023 Oct 5;13:16782. doi: 10.1038/s41598-023-43582-0 (PMC10556051; doi:10.1038/s41598-023-43582-0)
Supplement: Supplementary file 3 — Supplementary Table S2. [file 41598_2023_43582_MOESM3_ESM.docx]

**Supplementary Tab.S1** **Data set division and difference analysis between groups**

| Variables | Total  (n = 139) | Train  (n = 84) | Validation  (n = 55) | p |
| --- | --- | --- | --- | --- |
| CTLA4, n (%) |  |  |  | 1 |
| Low | 56 (40) | 34 (40) | 22 (40) |  |
| High | 83 (60) | 50 (60) | 33 (60) |  |
| Age, n (%) |  |  |  | 0.682 |
| ~59 | 64 (46) | 37 (44) | 27 (49) |  |
| 60~ | 75 (54) | 47 (56) | 28 (51) |  |
| Gender, n (%) |  |  |  | 1 |
| Female | 34 (24) | 21 (25) | 13 (24) |  |
| Male | 105 (76) | 63 (75) | 42 (76) |  |
| Primary_tumor_site, n (%) |  |  |  | 0.901 |
| Larynx | 34 (24) | 20 (24) | 14 (25) |  |
| Oral Cavity | 84 (60) | 52 (62) | 32 (58) |  |
| Oropharynx/Hypopharynx | 21 (15) | 12 (14) | 9 (16) |  |
| HPV_status, n (%) |  |  |  | 0.382 |
| Negative | 15 (11) | 7 (8) | 8 (15) |  |
| Positive/Unknown | 124 (89) | 77 (92) | 47 (85) |  |
| Grade, n (%) |  |  |  | 0.964 |
| G1/G2 | 97 (70) | 58 (69) | 39 (71) |  |
| G3/G4/GX | 42 (30) | 26 (31) | 16 (29) |  |
| Perineural_invasion, n (%) |  |  |  | 0.41 |
| NO | 48 (35) | 26 (31) | 22 (40) |  |
| Unknown | 49 (35) | 33 (39) | 16 (29) |  |
| YES | 42 (30) | 25 (30) | 17 (31) |  |
| Radiotherapy, n (%) |  |  |  | 0.626 |
| NO | 68 (49) | 43 (51) | 25 (45) |  |
| YES | 71 (51) | 41 (49) | 30 (55) |  |
| T_stage, n (%) |  |  |  | 0.424 |
| T1/T2 | 42 (30) | 28 (33) | 14 (25) |  |
| T3/T4/TX/Unknown | 97 (70) | 56 (67) | 41 (75) |  |
| N_stage, n (%) |  |  |  | 1 |
| N0 | 54 (39) | 33 (39) | 21 (38) |  |
| N1/N2/N3/NX/Unknown | 85 (61) | 51 (61) | 34 (62) |  |
| M_satge, n (%) |  |  |  | 0.24 |
| M0 | 66 (47) | 36 (43) | 30 (55) |  |
| M1/MX/Unknown | 73 (53) | 48 (57) | 25 (45) |  |
| Chemotherapy, n (%) |  |  |  | 1 |
| NO | 96 (69) | 58 (69) | 38 (69) |  |
| YES | 43 (31) | 26 (31) | 17 (31) |  |
| OS, n (%) |  |  |  | 0.092 |
| 0 | 88 (63) | 48 (57) | 40 (73) |  |
| 1 | 51 (37) | 36 (43) | 15 (27) |  |
| OS.time, Median (Q1,Q3) | 29.63 (14.42, 49.07) | 28.67 (14.95, 48.97) | 29.63 (13.6, 49.48) | 0.935 |
