# Supplementary material for: Noninvasive radiomic analysis of enhanced CT predicts CTLA4 expression and prognosis in head and neck squamous cell carcinoma
Source: Sci Rep. 2023 Oct 5;13:16782. doi: 10.1038/s41598-023-43582-0 (PMC10556051; doi:10.1038/s41598-023-43582-0)
Supplement: Supplementary file 4 — Supplementary Legends. [file 41598_2023_43582_MOESM4_ESM.docx]

**Supplementary figure legends**

Supplementary Fig. S1. Correlation heatmap between RS and immune genes. RS is the abbreviation for radiomics score. The lower left corner represents the correlation coefficient, and the upper right corner represents the p-value.

**Supplementary table legends**

Supplementary Tab. S1. The table shows details of radiomic feature extraction. A total of 107 features were extracted.

Supplementary Tab. S2. The table shows data set division and difference analysis between groups. OS in the table is the abbreviation for Overall Survival. The p-value of each variable in the analysis of differences between groups is >0.05.
